# Multiple merger coalescent inference of effective population size

**DOI:** 10.1098/rstb.2023.0306

**Published:** 2025-02-20

**Authors:** Julie Zhang, Julia A. Palacios

**Affiliations:** ^1^ Department of Statistics, Stanford University, CA 94305, USA; ^2^ Department of Biomedical Data Science, Stanford University, CA 94305, USA

**Keywords:** Multiple mergers coalescent, Gaussian processes, Lambda-coalescent, Beta-coalescent

## Abstract

Variation in a sample of molecular sequence data informs about the past evolutionary history of the sample’s population. Traditionally, Bayesian modelling coupled with the standard coalescent is used to infer the sample’s bifurcating genealogy and demographic and evolutionary parameters such as effective population size and mutation rates. However, there are many situations where binary coalescent models do not accurately reflect the true underlying ancestral processes. Here, we propose a Bayesian non-parametric method for inferring effective population size trajectories from a multifurcating genealogy under the Λ-coalescent. In particular, we jointly estimate the effective population size and the model parameter for the Beta-coalescent model, a special type of Λ-coalescent. Finally, we test our methods on simulations and apply them to study various viral dynamics as well as Japanese sardine population size changes over time. The code and vignettes can be found in the phylodyn package.

This article is part of the theme issue ‘“A mathematical theory of evolution”: phylogenetic models dating back 100 years’.

## 1. Introduction

In phylodynamic inference, variation in a sample of molecular sequence data is used to learn about the past ancestral history of a sample, usually represented by a bifurcating genealogy. Kingman’s coalescent is then typically used as a prior model on the genealogy, parameterized in terms of a parameter of interest called the effective population size $N_{e}(t)$ [1,2]. It has been shown that Kingman’s coalescent is a good approximation to the distribution of the sample’s ancestry under several underlying population dynamics [3]. However, Kingman’s coalescent assumes the population variance in the number of offspring is sufficiently small such that at most two lineages merge at a time. Such bifurcating tree models may not fit all population dynamics of interest. This can occur in the study of infectious disease dynamics in the presence of superspreader events. Examples include tuberculosis [4] and wild type polio [5]. The same situation arises in highly fecund species populations, where certain individuals are capable of reproducing offspring on the order of the population size; this is known as reproductive skew or sweepstakes reproduction [6,7]. Some marine species undergo sweepstakes reproduction, such as the Japanese sardine [8], Pacific oysters [9] and Korean seaweed [10]. Understanding the phylodynamics of these populations may have impacts on the economy and the environment. Multifurcating trees can also arise in populations undergoing strong positive selection [11–13] and in large sample sizes from small populations [14]. Here we assume neutral evolution without recombination. In addition, we only consider multifurcating trees without simultaneous mergers, a situation that would arise in diploid populations with sweepstake reproduction [15].

The Λ-coalescent is a multiple merger coalescent model (MMC) proposed as a generalization to binary coalescent processes [16,17]. The Λ-coalescent includes Kingman’s coalescent, the Beta-coalescent [18] and the Psi-coalescent [19] as special cases. In particular, the Beta-coalescent is a one-parameter model that was shown to be the ancestral limit of a sample obtained from the Cannings population model with a heavy tail offspring distribution and constant population size [20,21]. In Hoscheit & Pybus [22], the authors extended the Beta-coalescent model to allow variable effective population sizes and heterochronous sampling, i.e. when tips have different dates. The effective population size under Kingman’s coalescent can be interpreted as the population size under the Wright–Fisher model that has the same genetic drift as the population under study [3]. Under the Beta-coalescent, a similar interpretation holds for a population that evolves under the Cannings population model with a specific offspring distribution. See Eldon [7] for a recent review of MMC models and Korfmann *et al.* [23] for modelling recombining genealogies with MMC.

Most applications of the Λ-coalescent aimed to distinguish between population growth, such as exponential growth, and multiple merger genealogy from summaries of molecular data including the site frequency spectrum (SFS) [24–28]. A recently proposed method estimates the base measure Λ that describes the coalescent rates from the SFS [29]. However, all these methods assume constant effective population size or a fixed growth model and the infinite-sites mutation model. Instead, a method for inferring effective population sizes under the Beta-coalescent from a given multiple merger genealogy is proposed in Hoscheit & Pybus [22]. Although this method ignores genealogical uncertainty, it opens the door to applications in which an estimated genealogy is obtained under any mutation model (for example via maximum likelihood estimation). The authors propose an extension of the classic skyline plot [30] for inferring the effective population size; however, there is no joint inference of effective population size and the characteristic measure that describes coalescent rates.

In this article, we propose a method for joint inference of the effective population size $N_{e}(t)$ and the characteristic coalescent measure under the Beta-coalescent. We expand upon the work of Palacios & Minin and Lan *et al.* [31,32] and develop a Bayesian non-parametric phylodynamic approach that relies on Gaussian Markov random field priors on $N_{e}(t)$. We evaluate the performance of proposed methods on simulations and apply them to analyse two infectious diseases. Finally, we re-analyse the reproductive skew hypothesis in Japanese sardine populations [8] using our methods. Those who are interested in examining case studies can focus on §§4 and 5.

## 2. Background on Λ-coalescent

The standard Kingman’s n-coalescent is a backward-in-time Markov jump chain on binary partitions of [n]={1,…,n} whose full realization is a genealogy of n individuals. The process starts with n singleton lineages at time 0. At each step, two lineages are chosen uniformly at random to coalesce, continuing until there is a single lineage at the root [1]. The coalescent holding times are exponentially distributed with rate (A(t)2), when there are A(t) ancestral lineages at time t. Kingman’s coalescent has also been extended to incorporate variable population size, also termed the effective population size $N_{e}(t)$ [2], and samples collected at different times (heterochronous sampling) [33]. Here, $N_{e}(t)$ is interpreted as the size of an ideal population that exhibits the same level of genetic drift under a standard Wright–Fisher population model [34], and can be thought of as a relative measure of genetic diversity over time. The effective population size affects the coalescent times: larger $N_{e}(t)$ implies smaller coalescent rates and longer time until coalescence.

The Λ-coalescent is a generalization of the standard coalescent that allows for multiple mergers [16,17]. The process is a Markov jump chain on partitions of [n]={1,…,n} whose full realization is a multiple merger genealogy of n individuals. The process starts with n singleton lineages at time 0. At each step, two or more lineages are chosen to coalesce, continuing until there is a single lineage at the root (figure 2). Here, k≥2 lineages merge at rate $\lambda_{A(t),k}$, where A(t) is the number of extant lineages at time t. The coalescent holding times are exponentially distributed with rate λA(t)=∑k=2A(t)(A(t)k)λA(t),k, also called the total coalescent rate. Conditional on a coalescent event at time t when there are A(t) lineages, the distribution of the block (or multiple merger) size is


(2.1) P(X=k)=(A(t)k)λA(t),k∑i=2A(t)(A(t)i)λA(t),i,2≤k≤A(t).

The k lineages in the block are then chosen uniformly at random among the A(t) lineages. The rate $\lambda_{b,k}$ at which a specific k-block of lineages merges when there are b lineages is defined to be


(2.2) $$\begin{array}{lcr} & \lambda_{b,k}=\int_{0}^{1}x^{k-2}(1-x{)}^{b-k}\Lambda (dx), & \end{array}$$

where Λ is a measure on [0,1].

It is shown in Pitman [16] that any simple Markovian MMC process that satisfies exchangeability of the lineages and consistency of the merger rates defined by $\lambda_{b,k}=\lambda_{b+1,k}+\lambda_{b+1,k+1}$ must be the Λ-coalescent. The intuition behind the consistency condition is as follows. Suppose we have a specific k-block of lineages out of b lineages. If there are b+1 total lineages, then the extra lineage can coalesce with the k-block at rate $\lambda_{b+1,k+1}$ or the extra lineage does not coalesce with the k-block at rate $\lambda_{b+1,k}$, and so the additive property must hold. The Λ-coalescent can also be constructed as a Poisson point process on (0,1]×(0,∞) with intensity measure $\Lambda (dx)x^{-2}⊗dt$ (see [21] for a detailed theoretical construction). We can interpret points $\left(x_{i},t_{i}\right)$ from this Poisson point process as flipping a coin with probability $x_{i}$ of heads for each block and merging all blocks that are heads at time $t_{i}$.

Some special cases of the Λ-coalescent include:

— $\Lambda =\delta_{0}$, i.e. the point mass at 0: in this case, $\lambda_{b,k}=1$ for k=2 and corresponds to *Kingman’s coalescent*.
— $\Lambda =\delta_{1}$, i.e. the point mass at 1: in this case, then nothing happens for an exponential amount of time with mean 1, at which point all lineages coalesce and we get the *star-shaped coalescent*.
— Λ= Beta(2−α,α) for 0<α<2: in this case, $\lambda_{b,k}=\text{B}(k-\alpha ,\alpha +b-k)/\text{B}(2-\alpha ,\alpha )$ where $\text{B}(2-\alpha ,\alpha )=\frac{\Gamma (2-\alpha )\Gamma (\alpha )}{\Gamma (2)}$ and this is called the *Beta-coalescent*. A special case when α=1 is known as the *U*-coalescent or the *Bolthausen-Sznitman coalescent* and

λb,k=(k−2)!(b−k)!(b−1)!=[(b−1)(b−2k−2)]−1.

**Figure 1.**
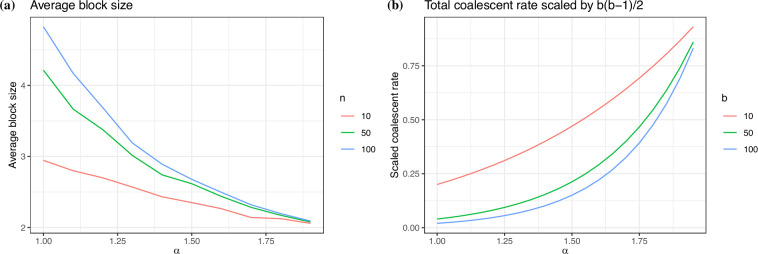
The effect of α in tree topology and coalescent times. (*a*) Average block size for different values of α and number of tips. (*b*) Total coalescent rates λb=∑k=2b(bk)λb,k when there are b lineages for different values of α, scaled by (b2), the rate under Kingman's coalescent.

The definition of the base measure Λ, and in particular the value of α in the Beta-coalescent, affects both the shape of the tree topology and the distribution of the coalescent times. To see this, in figure 1a, we plot the average block size for different values of α and number of tips n. We simulate 500 trees for each value of α and n and then compute the average block size across all trees and multiple merger events. We see the average block size decreases to 2, the binary tree, as α→2. The average block size also decreases with n, since trees with more tips are expected to have larger block sizes. In figure 1b, we show the total coalescent rate for different values of α and lineages b, divided by (b2), the total rate under Kingman’s coalescent. The coalescent rate increases exponentially with α. The smaller the value of α, the smaller the coalescent rate resulting in genealogies with longer branch lengths than with Kingman’s coalescent.

### (a) Heterochronous Λ-coalescent with variable $N_{e}(t)$

**Figure 2.**
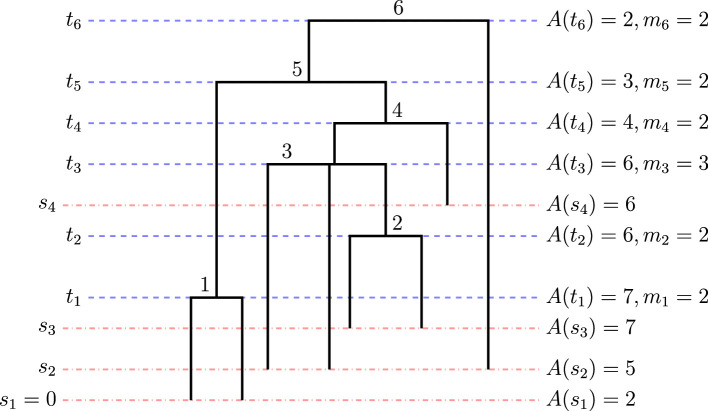
A multifurcating tree with eight total lineages and six coalescent events labelled with the coalescent times t, sampling times s, block sizes m and extant lineages A(t). Here, there are L=3 sampling times.

The Λ-coalescent was recently extended to accommodate heterochronous sampling and variable effective population size [22]. It is important to account for heterochronous sampling, especially for rapidly evolving organisms: the coalescent time distribution will be restricted since samples cannot coalesce before they have been sampled. Here, we assume that a rooted and timed multifurcating genealogy of n haploid samples is available to us, for example, the estimated genealogy obtained via maximum likelihood from a set of n observed molecular sequences. We further assume that sequences, at the tips of the genealogy, are collected at L different sampling times and that the sampling process is independent of the underlying population process. Let $n=(n_{\ell }{)}_{\ell =1:L}$ denote the number of samples collected at respective sampling times $s=(s_{\ell }{)}_{\ell =1:L}$, with $s_{1}=0$, $s_{j-1}<s_{j}$ for j=2,…,L and $n=\sum_{j=1}^{L}n_{j}$ is the total number of samples. In the genealogy, tuples of lineages merge backward in time into a common ancestor at coalescent times denoted by $t=(t_{1},\ldots ,t_{K})$, where K is the step at which the most recent common ancestor (MRCA) is reached (see figure 2).

Let $m=(m_{1},\ldots ,m_{k})$ denote the corresponding vector of block sizes at each coalescent time. The rate at which pairs of lineages coalesce depends on the number of lineages, the Λ measure and the effective population size $\left(N_{e}(t){)}_{t\geq 0}:=N_{e}(t\right)$. Finally, let the number of extant lineages at time t be given by $A(t)=\sum_{i=1}^{L}n_{i}1(s_{i}<t)-\sum_{k=1}^{K}(m_{k}-1)1(t_{k}<t)$. Under this model, the density of observed statistics m and t is:


(2.3) p(m,t∣n,s,Ne(t),Λ)=exp⁡(−∫0∞∑j=2A(u)(A(u)j)λA(u),jNe(u)du)∏k=1K(A(tk)mk)λA(tk),mkNe(tk).

The density corresponds to the density of a point process of coalescent events with rate λA(tk)=∑j=2A(tk)(A(tk)j)λA(tk),j when there are $A(t_{k})$ lineages at time $t_{k}$, and the probability of K coalescent events each being of size $m_{k}$:


(2.4) ∏k=1K(A(tk)mk)λA(tk),mkλA(tk).

Under this MMC framework, we ignore the effects of population structure, recombination and selection.

In this manuscript, we are interested in estimating the effective population size $N_{e}(t)$ and the α parameter in the Beta(2−α,α)−coalescent with α∈(0,2] because it has desirable properties. As mentioned above, it is the limiting distribution of a specific Cannings population model. This model also converges to Kingman’s coalescent model as α→2 from below. It was shown in Hoscheit & Pybus [22] that the α parameter in the Beta-coalescent model is indicative of the level of superspreading in an infectious population. The mathematical basis of the Beta-coalescent for α∈(0,1] does not change, so for the sake of implementation, we allow α∈(0,2] in parameter estimation. However, most simulation results will focus on Beta-coalescent trees generated with α>1.

Proposition 1 in appendix A.1 states that when there are A(t)=b lineages, the total Lambda-coalescent factor can be approximated as follows:


(2.5) λb=∑k=2b(bk)λb,k≈(b−1)(b2)α−1.

That is, the logarithm of the total coalescent rate when there are A(t)=b lineages is


$$\log\left(\int_{0}^{t}\frac{\lambda_{b}du}{N_{e}(u)}\right)\approx (\alpha -1)\;\log(b/2)+\log(b-1)+\log\left(\int_{0}^{t}\frac{du}{N_{e}(u)}\right).$$

Although we do not use this approximation for our inference methods, it provides intuition about the relationship between coalescent time distribution and its dependence on α.

Interpretation of $N_{e}(t)$ requires knowledge of the population’s variance in the number of offspring. In particular, under the Cannings population model, with variance of the number of offspring $c_{N}$, and assuming $c_{N}$ converges to 0 as N→∞, and the offspring distribution follows $\mathbb{P}(\nu_{1}>x)∼Cx^{-\alpha }$ for 1≤α<2 [20,35], then the coalescent process converges to the Beta-coalescent with


$$N_{e}(t)=\lim_{N\to \infty }\;\frac{N(\lceil x/c_{N}\rceil )}{1/c_{N}}.$$

In general, we assume populations whose effective population size is scaled in units of $1/c_{N}$ generations. We note that this factor depends on α in the case of the Cannings model; however, as long as $c_{N}$ remains constant over time, we can jointly estimate $N_{e}(t)$ and α for the Beta-coalescent and solve for N(t) if $c_{N}$ is known. Alternatively, the exact parametric form of $c_{N}$ could be incorporated directly in equation (2.3).

### (b) Estimation of $N_{e}(t)$ using a Gaussian Markov random field prior

We follow the approach developed in Palacios & Minin [31] and model $N_{e}(t)=\exp[\gamma (t)]$, where γ(t) is *a priori* an intrinsic Gaussian Markov random field (GMRF) [36], that is, γ(t) is a random piece-wise constant function with change points placed at a regular grid of D points $\left\{x_{1},...,x_{D}\right\}$. That is


$$N_{e}(t)=\sum_{d=1}^{D-1}\exp(\gamma_{d})1_{\left(x_{d},x_{d+1}\right]}(t)$$

and $(\gamma_{1},\ldots ,\gamma_{D-1})∼MVN(0,(\tau Q{)}^{-1})$, τ is the precision parameter with Gamma prior Gamma(0.001,0.001) and Q is the corresponding inverse covariance kernel of a random walk with boundary correction. For more technical details, see Palacios & Minin [31]. The integral in the exponent of equation (2.3) is then approximated by its Riemann sum. We adapted the implementation for binary trees in phylodyn [37] to the MMC with likelihood equation (2.3). Given m,s,t,n, and α, our implemented R function BNBR_Lambda () estimates posterior mean and 95% BCI of $N_{e}(t)$ by an integrated nested Laplace approximation (INLA) [38].

## 3. Methods

*Estimating* **α** *from tree topology only*: by the end of §2, we showed that α affects both the tree shape and the distribution of coalescent times. While coalescent times are the sufficient statistics for inferring $N_{e}(t)$ in the standard coalescent process, coalescent times and tree topology (i.e. merging block sizes) are the sufficient statistics for estimating $N_{e}(t)$ and α under the Beta-coalescent. However, it is possible that in some applications, there is no reliable estimation of coalescent times and it may be preferable to estimate α from the tree topology only. In this case, we propose to estimate α using just the block sizes m by maximizing the following pseudo-likelihood of the block sizes under the Beta(2−α,α) coalescent (recall equation (2.4)):


(3.1) α^BS=arg maxαP(m∣n,α)=arg maxα∏k=1K(A(tk)mk)λA(tk),mk(α)∑i=2A(tk)(A(tk)i)λA(tk),i(α).

We know there is information about α in both the block sizes and the coalescent times. However, we show in §4 that estimation of α based on tree topology only (block sizes) is quite accurate.

*Hybrid estimation of* **α** *and* : our first approach for estimating α and $N_{e}(t)$ from a multiple merger genealogy is a hybrid approach, where we iteratively update α and $N_{e}(t)$. We first initialize $\alpha^{\left(0\right)}={\hat{\alpha }}^{BS}$ to be the block-size only maximum likelihood estimator (MLE). Given α, we then use INLA, as described in §2.2, and update $N_{e}(t)$ by the posterior median. Given the current value of $N_{e}(t)$, we then find α by maximum likelihood estimation and iterate these last two steps until convergence.

*Joint posterior inference of* **$N_{e}(t)$** *and* : we place a GMRF prior on $\log\;N_{e}(t)$ as described in §2.2 with a Gamma prior on precision parameter τ and a uniform U(0,2) prior on α. We approximate $P(\alpha ,N_{e}(t),\tau |g)$ via a Metropolis-within-Gibbs algorithm, where g denotes the multifurcating genealogy. The precision parameter and the GMRF are usually highly correlated leading Markov chain Monte Carlo (MCMC) methods to have poor convergence and slow mixing. To solve this problem, we sample from the conditional distribution of $\log\;N_{e}(t)$ and τ given all other model parameters and genealogy, with the split Hamiltonian Monte-Carlo (sHMC) method adapted from Lan *et al.* [32]. To sample α from the full marginal


(3.2) $$P(\alpha |g,N_{e}^{\left(k\right)}(t),\tau )=\frac{P(g|N_{e}^{\left(k\right)}(t),\alpha )}{\int_{0}^{2}P(g|N_{e}^{\left(k\right)}(t),\alpha )d\alpha },$$

we discretize the parameter space of α∈[0,2] into intervals of length 0.005 and approximate the denominator of equation (3.2) by the Riemann sum. That is, we set $\alpha_{m}$ to be the midpoint of interval $I_{m}=\left[0.005m,0.005(m+1)\right]$ for m=1,...,400. The numerator of equation (3.2) is proportional to the likelihood of equation (2.3), and the proportionality constant cancels in the fraction. Then


$$P(\alpha \in I_{m}|g,N_{e}^{\left(k\right)}(t))\approx \frac{P(g|N_{e}^{\left(k\right)}(t),\alpha_{m})}{\sum_{i=1}^{400}P(g|N_{e}^{\left(k\right)}(t),\alpha_{i})}.$$

From this discrete distribution, we sample $I_{m}∼P(\alpha \in I_{m}|g,N_{e}^{\left(k\right)}(t)),\alpha^{\left(k+1\right)}∼Unif[I_{m}]$ to get our final update.

## 4. Results

We first test our methods on simulated data. We implement a function that can simulate a Beta-coalescent genealogy under variable effective population size and heterochronous sampling. We use the true genealogy as input in simulation studies. To evaluate their performance in estimating α, we use the mean squared error (MSE). For evaluating the performance in estimating the effective population size, we compute four metrics: coverage, bias, deviance and MSE at the grid points, with precise definitions in appendix A.2.

To see how well we can estimate α from tree topology alone (block size data), we generated 1000 realizations from the Beta-coalescent under different values of α,n. Table 3 in appendix A.3 shows the mean, bias and MSE of the estimated α using only the block size data. Overall, we see ${\hat{\alpha }}^{BS}$ underestimates α and the accuracy of the estimator increases with n and α. When α=1.5, the accuracy of this estimator is quite high for trees with 100 tips. When α=1.8, the results are accurate for trees with 50 tips.

**Figure 3.**
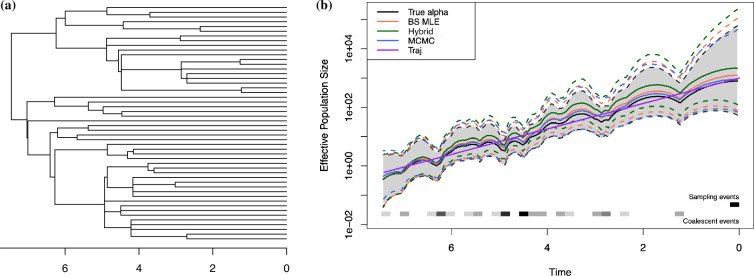
Comparison of different methods estimating exponential **$N_{e}(t)$**. (*a*) A simulated genealogy under the Beta-coalescent with α=1.5 and the exponential growth $N_{e}(t)$ trajectory. The genealogy has 39 internal nodes and 50 tips. (*b*) The effective population size trajectories reconstructed by different methods. Note the *y*-axis is plotted in log-scale. Solid lines are the median trajectories, and dotted lines are the 95% credible bands. The true trajectory is plotted in purple, and the solid black line and shaded band are the median and 95% credible band of the inferred $N_{e}(t)$ using the true α=1.5.

In the left plot in figure 3, we show one isochronous tree with 50 tips, generated with α=1.5 and exponential growth $N_{e}(t)=1000e^{-t}$. The block-size MLE is ${\hat{\alpha }}^{BS}\approx 1.615$ and the hybrid estimate is ${\hat{\alpha }}^{H}\approx 1.759$. The posterior median and mean of α inferred from MCMC are 1.594 and 1.585, respectively, which are the closest to the true α value. Figure 11 in Appendix A.3 shows the posterior distribution of α from MCMC. The right plot in figure 3 depicts the reconstructed effective population size trajectories and their 95% credible regions estimated with each method, as well as the true trajectory. Table 2 in appendix A.3 details the performance measures calculated for inferred $N_{e}(t)$ with each method. We see that inferring $N_{e}(t)$ using the true α has the lowest bias, deviance and MSE, while out of the three proposed methods, MCMC performs the best and hybrid estimation the worst. This is expected since the ${\hat{\alpha }}^{H}$ is the worst estimate. In appendix A.3 figure 12, we show trace plots of α and $\log\;\;N_{e}(t)$ at a particular value of t to demonstrate well-mixing.

To evaluate the overall performance of our methods for the joint inference of α and $N_{e}(t)$, we simulated 50 trees for each combination of α∈{1,1.5,1.8}, n∈{20,50,100} with three sampling schedules: (i) all sampled at time 0 (isochronous); (ii) heterochronous with two sampling times, 50% split; and (iii) heterochronous with four sampling times, split 50, 30, 10 and 10% across the four times. We also considered three effective population size trajectories: (i) uniform (constant): $N_{e}(t)=100$; (ii) exponential growth: $N_{e}(t)=1000e^{-t}$; and (iii) boom–bust: $N_{e}(t)=1000e^{-|t-1|}$.

For the MCMC method, we run 20 000 iterations discarding the first 10% as burn-in. For each tree, we obtain the following set of results for α: the block-size ${\hat{\alpha }}^{BS}$, the estimate from the hybrid method ${\hat{\alpha }}^{H}$ and a posterior distribution of α estimated via MCMC. We also infer the effective population size at a regular grid of 100 points using INLA and the true α value, the two estimates ${\hat{\alpha }}^{BS}$ and ${\hat{\alpha }}^{H}$, as well as the posterior distribution of $N_{e}(t)$ obtained by MCMC.

Figure 13 in appendix A.3 shows the boxplots of estimated α values for each simulated genealogy with three big blocks corresponding to the three $N_{e}(t)$ scenarios considered. The posterior median is used as the final α estimate in the MCMC case. Each plot corresponds to different α,n combinations, with the number of tips n varying across rows and α values varying across columns. The three different sampling schedules are indicated by the colour of the boxplots. The black line shows the true α value. First, we see the results do not vary much across the different sampling times, which is a good sign of robustness, and so going forward will pool results from the three different sampling times together. We also see that accuracy increases with α and the number of tips n.

MCMC is the top performing method according to deviance for estimating α for over 40% of the simulations, with blocksize MLE and hybrid estimation having similar performances (see table 4 in appendix A.3). Table 1 shows the summary of performance statistics for estimating $N_{e}(t)$ (with the different sampling times combined). The value in italics is the best performing (largest coverage, smallest deviance and smallest MSE) out of the three methods. In terms of coverage, the MCMC method has the best performance; however, all methods in general have good coverage. In terms of median deviance and mean MSE, the MCMC method is highly superior for the exponential and boom-and-boost trajectories; however, we see the block-size MLE performs the best for the uniform trajectory, with the hybrid method not far behind. One possible explanation is a lack of identifiability in the case of constant $N_{e}$: scaling α by c and $N_{e}$ be 1/c would result in the same likelihood.

**Table 1.** Mean coverage, median deviance and median MSE of estimated $N_{e}(t)$ for each trajectory and each method. The method with the best performance per N,α and trajectory combination is shown in italics.

			coverage			deviance			MSE		
	N	method	unif.	exp.	BB	unif.	exp.	BB	unif.	exp.	BB
α=1	20	BS MLE	0.92	0.96	0.95	*0.4*	1.23	1.57	*19.34*	117.14	287.95
		hybrid	0.91	0.84	0.84	0.55	3.76	5.03	31.7	1796.08	8197.65
		MCMC	*0.99*	*0.98*	*0.98*	0.54	*0.8*	*0.81*	34.59	*94.34*	*118.74*
	50	BS MLE	0.9	0.94	0.93	0.37	0.74	0.82	15.14	78.4	156.55
		hybrid	0.9	0.76	0.79	*0.356*	2.463	2.937	*15.101*	627.103	2389.601
		MCMC	*0.97*	*0.97*	*0.97*	0.42	*0.61*	*0.71*	25.6	*74*	*132.66*
	100	BS MLE	0.89	0.95	0.97	*0.32*	0.56	0.63	*12.49*	*42.99*	93.95
		hybrid	0.88	0.72	0.76	0.35	2.21	2.66	15.02	671.61	2454.44
		MCMC	*0.98*	*0.99*	*0.98*	0.393	*0.5*	*0.52*	20.06	49.37	*88.1*
α=1.5	20	BS MLE	0.95	0.97	0.96	*0.32*	0.79	0.88	*11.36*	63.39	130.03
		hybrid	0.95	0.94	0.95	0.33	1.28	1.67	11.6	121.85	677.61
		MCMC	*0.99*	*0.97*	*0.98*	0.33	*0.54*	*0.57*	14.22	*63.44*	*85.45*
	50	BS MLE	0.95	0.97	0.96	*0.26*	0.66	0.68	*7.47*	60.97	125.58
		hybrid	0.95	0.94	0.95	0.27	1.05	1.12	8.13	115.94	497
		MCMC	*0.98*	*0.99*	*0.98*	0.34	*0.58*	*0.57*	14.71	*52.19*	*85.16*
	100	BS MLE	0.95	0.94	0.95	*0.21*	0.43	0.46	*5.24*	36.06	50.33
		hybrid	0.95	0.85	0.87	0.21	0.74	0.81	5.33	71.44	193.77
		MCMC	*0.99*	*0.98*	*0.98*	0.28	*0.41*	*0.41*	9.84	*34.78*	*49.26*
α=1.8	20	BS MLE	0.98	0.98	0.98	*0.23*	0.66	0.74	6.44	60.77	153.67
		hybrid	0.99	0.97	0.98	0.24	0.74	0.86	*6.3*	*56.85*	207.42
		MCMC	*1*	*0.98*	*0.99*	0.34	*0.5*	*0.57*	14.7	59.86	*86.87*
	50	BS MLE	0.98	0.98	0.98	*0.2*	0.44	0.49	*4.25*	36.24	81.19
		hybrid	0.98	0.98	0.98	0.22	0.56	0.64	5	42.94	129.35
		MCMC	*0.99*	*0.99*	*0.99*	0.29	*0.41*	*0.41*	10.25	*27.56*	*56.11*
	100	BS MLE	0.97	0.96	0.96	*0.17*	0.43	0.43	3.6	23.18	37.24
		hybrid	0.97	0.96	0.97	0.16	0.48	0.47	*3.27*	25.03	52.15
		MCMC	*0.99*	*0.97*	*0.98*	0.24	*0.4*	*0.39*	7.7	*22.89*	*31.76*

With regard to the computational cost of the methods, one run of the MCMC method (for 20 000 iterations) on a multifurcating tree with 100 tips takes approximately 15 minutes on one CPU node, while the BS MLE method takes less than 10 seconds, and the hybrid method takes 3 minutes. Computational complexity of all methods is roughly linear in n, the number of tips.

## 5. Applications

We apply our methods to two infectious disease examples and one animal population, where multifurcating viral phylogenies may occur due to superspreading events or skewed reproduction. All data used here can be downloaded directly from the open source dashboard Nextstrain [39] or from the GenBank database [40]. We generated point estimates of the genealogies ignoring uncertainty and the quality of the estimation procedure. Our methods are not robust against misestimations of the genealogy (see appendix A.4), and incorporation of genealogical uncertainty is still an open problem.

### (a) Respiratory syncytial virus (RSV)

RSV is a respiratory illness transmitted via droplets that mostly affects children and immunocompromised individuals. Globally in 2019, there was an estimated 33 million RSV-associated acute lower respiratory infection episodes (ARTI) in children under the age of five [41]. These ARTI episodes mostly result in hospitalizations and healthcare burdens, and current surveillance efforts are not well-developed, especially in lower income or lower-middle income countries such as those in southeast Asia [42,43]. Research also suggests that RSV may be seasonal, with more than 75% of cases within five months, mainly occurring during the rainy season in tropical countries [44,45]. In terms of the virology, RSV is a negative-sense, single-stranded RNA virus with 10 genes that encode 11 proteins [46]. In particular, two major surface proteins (F and G glycoproteins) control viral attachment and are the primary antibody targets, with the G protein being highly variable. There are also two antigenic subtypes based on the reaction of F and G proteins to monoclonal antibodies: RSV-A and RSV-B. Research shows that RSV-A is more prevalent and virulent than RSV-B, making it more concerning in terms of public health [47,48].

**Figure 4.**
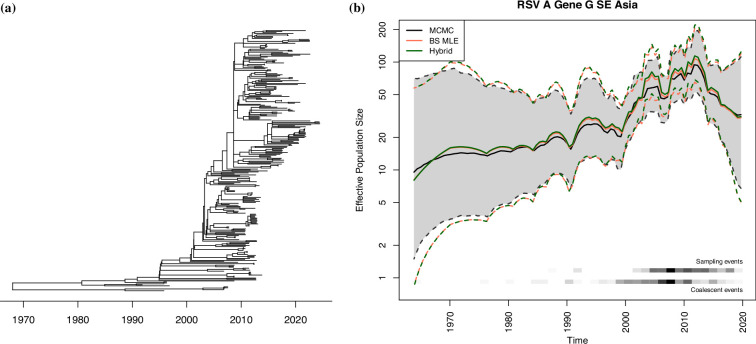
(*a*) A dated genealogy generated using 266 RSV-A sequences from Nextstrain. (*b*) The reconstructed effective population size trajectories for RSV-A sequences in south and southeast Asia. Note the y-axis is plotted in log-scale. Solid lines are the median trajectory, and dotted lines are the 95% credible band.

Here, we consider the genealogy shown in figure 4a estimated from 266 RSV-A molecular sequences^1^. We accessed the webpage on April 9, 2024. The block-size MLE is ${\hat{\alpha }}^{BS}=1.718$ and the hybrid estimate is ${\hat{\alpha }}^{H}=1.734$, and the MCMC posterior median and mean are 1.698 and 1.696. The inferred effective population size trajectories are shown in figure 4b. We see a rather steady increase until around 2010, with some oscillations indicative of the seasonality of RSV. The credible intervals obtained with MCMC are overall narrower than those obtained with the other two methods.

### (b) *Enterovirus* D68

First discovered in 1962, *Enterovirus* D68 is a non-polio enterovirus that causes respiratory illnesses. It is spread person-to-person via respiratory secretions and mainly affects children. *Enterovirus* D68 has been found to be associated with acute flaccid myelitis (AFM), a muscle weakness condition which can lead to paralysis, but not caused by polio [52,53]. There is no treatment or vaccine and so large outbreaks are important public health concerns. Prior to 2014, there were only 699 confirmed cases, but since then, outbreaks in the United States, Canada, Europe and Asia have regularly occurred. Over 2000 cases were confirmed worldwide in 2014 alone [54]. Since diagnoses can only be confirmed via laboratory testing, these case counts are likely to be underestimated. Cases are also seasonal, occurring mainly from August to October, with the US having had outbreaks on a two-year cycle in 2014, 2016 and 2018 [55,56].

**Figure 5.**
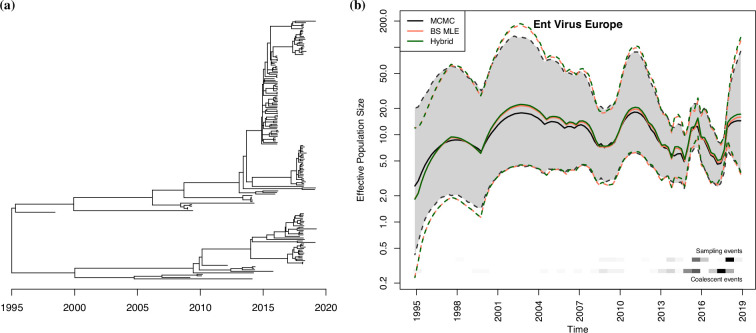
(***a***) A dated phylogeny generated using 171 *Enterovirus* D68 sequences from Nextstrain. (*b*) The reconstructed effective population size trajectories for *Enterovirus* D68 sequences in Europe. Note the y-axis is plotted in log-scale. Solid lines are the median trajectory, and dotted lines are the 95% credible band.

Post-COVID re-opening saw an outbreak of 139 cases in eight European countries between July and October 2021 [57]. To investigate the spread of *Enterovirus* D68 in Europe prior to COVID, we analyse a genealogy (figure 5a) inferred from 171 European sequences^2^ sampled between 1998 and 2019. Most samples are of children under the age of 5 and belong to clade B3, which was the dominant strain of the 2016 *Enterovirus* D68 outbreak in the US [58]. The block-size MLE is ${\hat{\alpha }}^{BS}=1.741$, the hybrid estimate is ${\hat{\alpha }}^{H}=1.764$. The MCMC posterior median and mean from MCMC are 1.732 and 1.728. The inferred effective population size trajectories are depicted in figure 5b. We see an oscillatory pattern, with a peak prior to 1998, then rather constant until a decrease in 2008 and then a peak around 2012. Then, we see peaks around 2015 and 2019 which is consistent with enterovirus outbreaks observed clinically, if we account for delays in sequencing. Again, the block-size MLE method and hybrid method give slightly higher estimates than MCMC. The credible intervals for MCMC are slightly narrower than the other two methods, but all three give similar results.

### (c) Japanese sardine populations

Three hypotheses have been postulated as possible explanations for an excess of low-frequency mutations in many marine species, such as sardines. The first is that populations experienced a population bottleneck followed by a period of expansion during and after the Last Glacial Maximum. The second hypothesis states that species experienced natural selection and selective sweeps. The third is the reproductive skew hypothesis: there exists high variation in the number of offspring produced by many individuals which is then reflected in the genetic diversity. The authors in Niwa *et al.* [8] analysed 106 mitochondrial DNA sequences of Japanese sardine (*Sardinops melanostictus*) collected in 1990, where they conclude the data strongly support the presence of multiple mergers. They assumed the Beta(2−α,α)−coalescent model as the underlying MMC model and used an importance sampling scheme with the infinite-sites mutation model to reject the hypothesis of α=2 [59], with α estimated to be approximately 1.3 by maximum likelihood. Further results in their analysis reject the demographic expansion hypothesis using site-frequency spectrum and pairwise nucleotide differences. Understanding the presence of reproductive skew is important in order to better estimate the current and historical effective population size of marine species.

**Figure 6.**
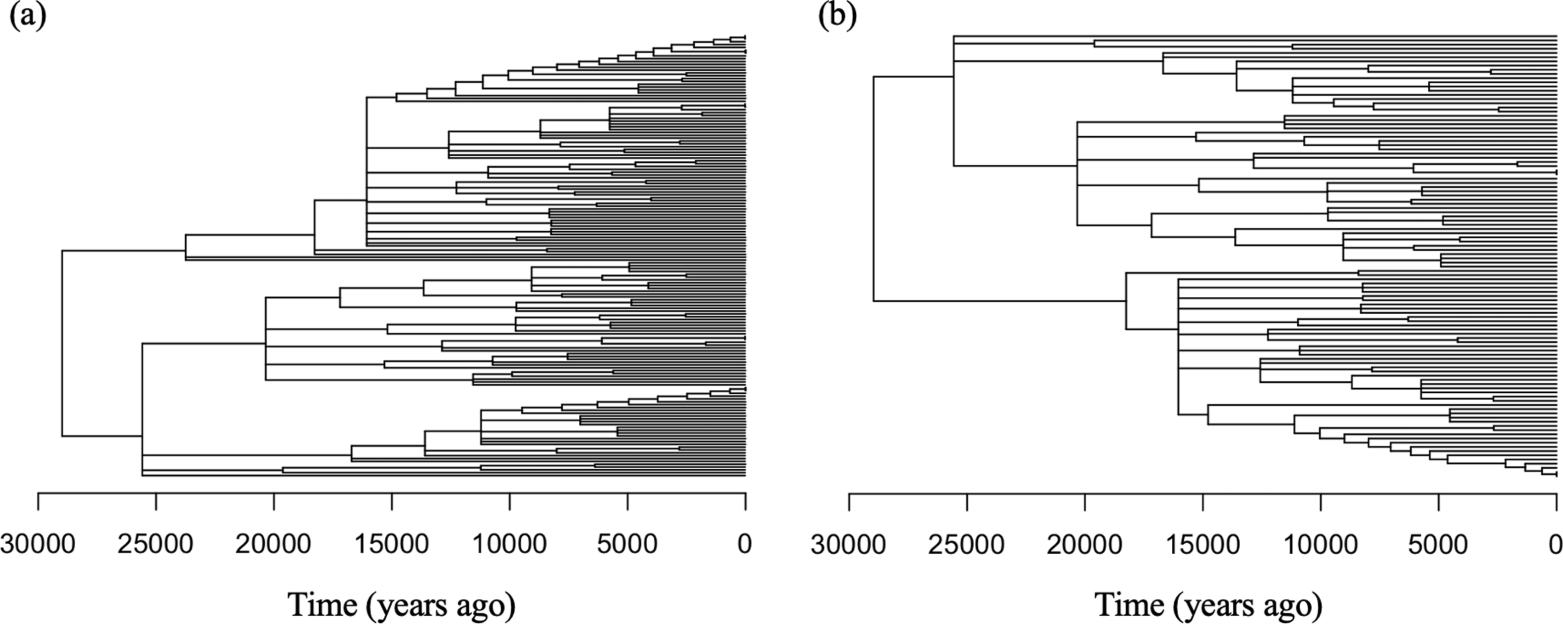
(*a*) A dated phylogeny generated using all *Sardinops melanostictus* sequences. There are 156 tips and 95 internal nodes. (*b*) The subtree constructed of only sequences sampled in 1990: 106 tips and 61 internal nodes.

We accessed the same mtDNA sequences from GenBank (accession nos LC031518–LC031673) and re-analysed the reproductive skew hypothesis with our methods. Out of the 156 total sequences, 106 were collected in 1990 and used in both of the previous articles for analysis, while the other 50 sequences, collected between 2010 and 2012 by the same authors were not included in the original study. We first used MAFFT to align the sequences of about 1200 bp of length, then used IQ-Tree v. 1.6.12 and TreeTime to infer dated, multifurcating trees via maximum likelihood [49–51]. Again, branches of length zero were collapsed. We fix the mutation rate in TreeTime to be $2\times 10^{-7}$ per site per year. The related Indian oil sardine, *Sardinella longiceps*, estimated a mutation rate of $1\times 10^{-7}$ per site per year with a strict molecular clock [60]. Figure 6a,b shows the inferred trees using 156 sequences and the subtree containing only sequences from 1990, respectively. The dated root is inferred to be around 29 000 years ago, which roughly coincides with around the time of the last glacial maximum.

**Figure 7.**
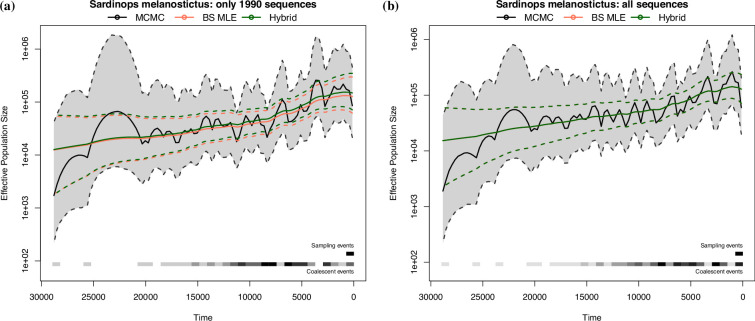
The reconstructed effective population size trajectories for (*a*) the 106 sequences sampled in 1990 and (*b*) all *Sardinops melanostictus* sequences. Note the y-axis is plotted in log-scale. Solid lines are the median trajectory, and dotted lines are the 95% credible band.

We first estimate model parameters from the subtree with only sequences from 1990. The block-size MLE is ${\hat{\alpha }}^{BS}=1.3498$, the hybrid estimate is ${\hat{\alpha }}^{H}=1.3855$, and the MCMC posterior median and mean are 1.319 and 1.317, respectively. These inferred α values are largely consistent with those reported in Niwa *et al.* [8] that used an alternative method. The inferred effective population size trajectories depicted in figure 7a show a slow increase in $N_{e}(t)$ over time; however, a constant trajectory falls within the 95% credible intervals of the MCMC posterior. The MCMC estimates are less smooth than the block-size MLE and the hybrid estimates. Analysing all sequences, we obtain the block-size MLE ${\hat{\alpha }}^{BS}=1.4119$, the hybrid estimate is ${\hat{\alpha }}^{H}=1.4124$ and the MCMC posterior median and mean are 1.3686 and 1.3659, respectively. Adding in extra data has reduced the variability in the MCMC method and also increased the inferred α values. The inferred effective population size trajectories are shown in figure 7b. Note the block-size MLE trajectory is covered by the hybrid estimate one because their α values are very close, but they are not identical. The inferred trajectories with all sequences are very similar to those inferred from only the sequences collected in 1990.

In Matuszewski *et al.* [26], the authors used site frequency spectrum (SFS)-based maximum likelihood methods under the ψ-coalescent and exponential population growth to jointly investigate reproductive skew and population expansion hypotheses. The ψ-coalescent is a Λ-coalescent with measure $\Lambda =\delta_{\psi },\psi \in \left[0,1\right]$ and is the limiting distribution of an extended, discrete-time Moran model [19]. The authors estimate $\hat{\psi }=0.46$, indicating reproductive skew, but no exponential population growth. Overall, our results agree with this study, except that our detailed inference of $N_{e}(t)$ shows a slow increase over time.

**Figure 8.**
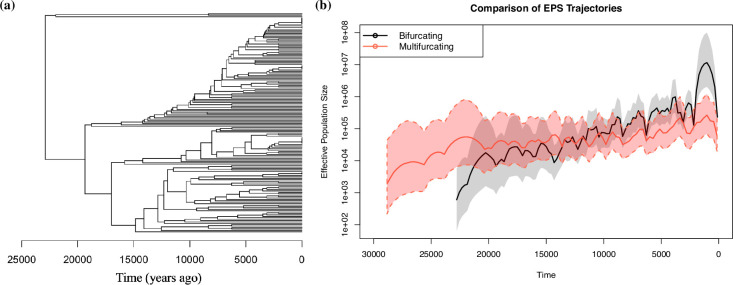
(***a***) The serially sampled UPGMA binary genealogy of all Japanese sardine sequences. (***b***) Comparison of the reconstructed $N_{e}(t)$ using BNPR for the binary UPGMA and the MCMC $N_{e}(t)$ for the multifurcating tree in figure 6a. Note that the $T_{MRCA}$ for the multifurcating tree in figure 6a is around 29 000 years versus 23 000 years for the binary genealogy shown in (*a*), which is why the inferred $N_{e}(t)$ profiles have different ranges for t.

Finally, we compare the inferred $N_{e}(t)$ trajectories under a Λ-coalescent model versus the Kingman’s variable $N_{e}(t)$ model. We use the methodology from Drummond & Rodrigo [61] to reconstruct the UPGMA binary genealogy of serial samples under a fixed molecular clock with Poisson mutations. The implementation can be found in the phylodyn package. We used the BNPR function in phylodyn to estimate $N_{e}(t)$ using the INLA approximation [31]. Figure 8 shows the inferred binary tree with a root date of around 23 000 years ago and the comparison of estimated $N_{e}(t)$ trajectories. The growth of $N_{e}(t)$ is much steeper under the bifurcating model than the multifurcating model, which suggests the inadequacy of the bifurcating model given previous work.

## 6. Discussion

**Figure 9.**
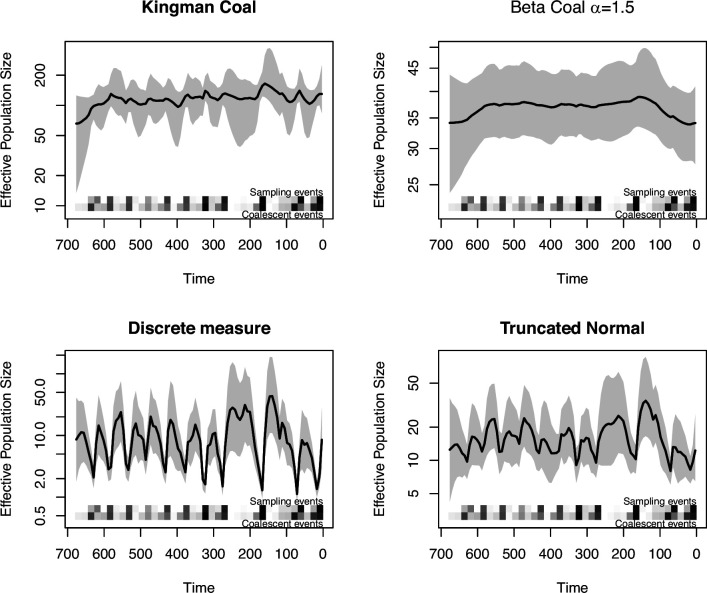
An example of inference of effective population size of a phylogenetic tree using BNPR under various coalescent models.

**Figure 11.**
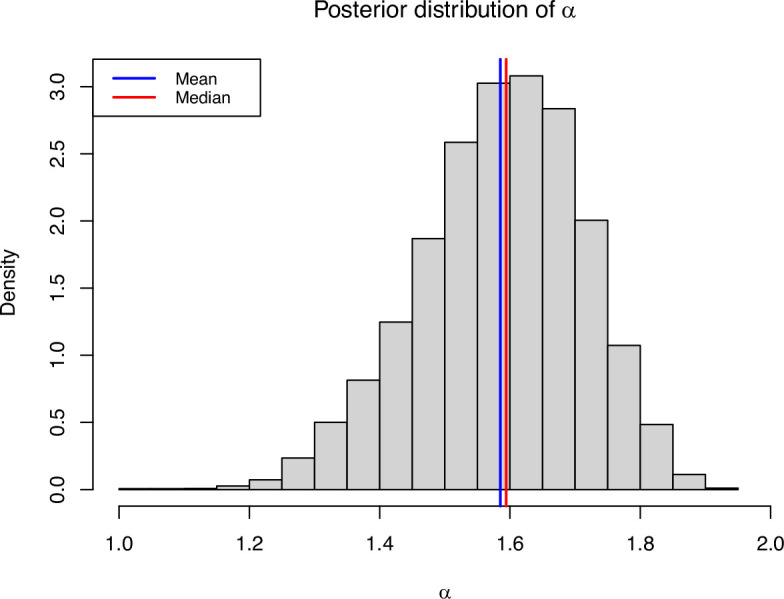
Posterior distribution of **α** for the tree in figure 3. The blue and red lines are the mean and median values, respectively.

**Figure 12.**
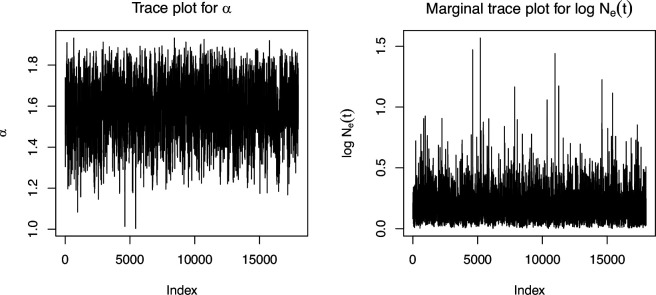
Trace plots for the MCMC chain for the tree in figure 3. The marginal trace plot for $\log\;N_{e}(t)$ is taken at t≈5.23, which is the 70th grid point.

**Figure 13.**
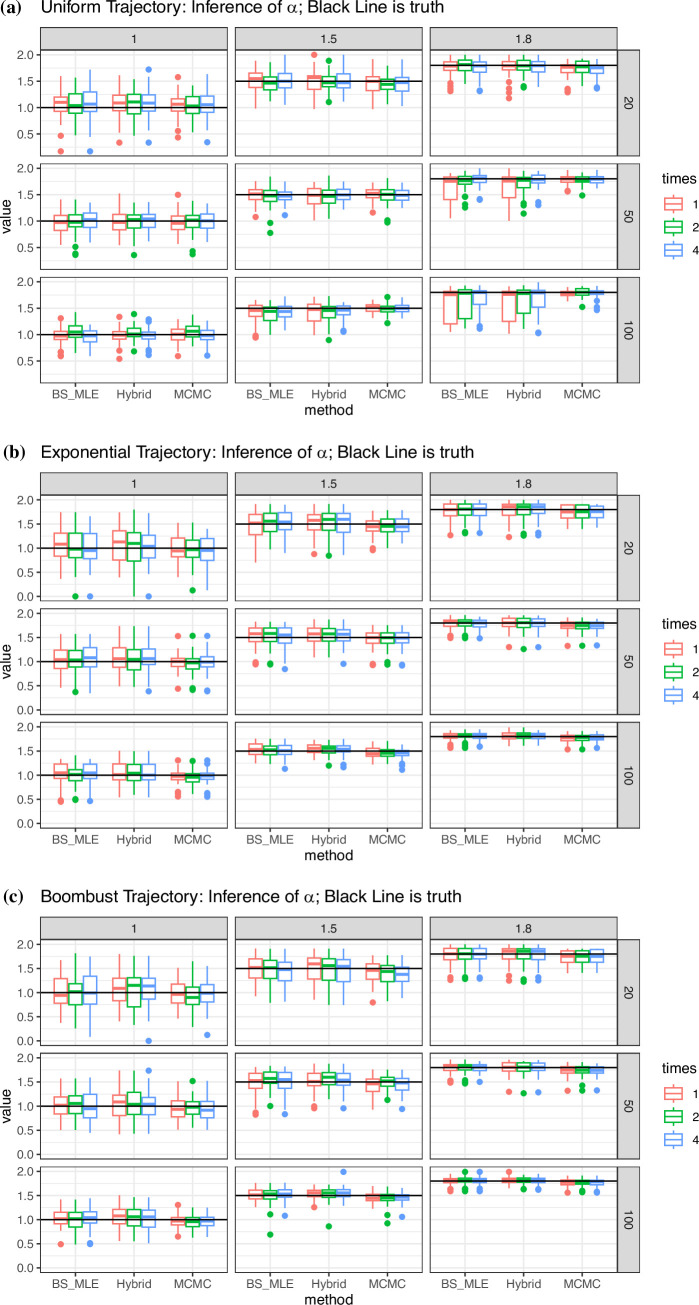
Boxplots of inferred α values from simulations with trees generated from (*a*): uniform trajectory, (*b*): exponential trajectory, (*c*): boombust trajectory. Each row is a fixed N; each column is a fixed α; and each colour is a sampling time. The black line is the true α.

In this manuscript, we propose three Bayesian non-parametric methods to jointly estimate the effective population size and α parameter from a multifurcating tree under the Beta(2−α,α)−coalescent model. Two of the proposed approaches iterate estimation between α and $N_{e}(t)$, and the third approach approximates the posterior distribution of parameters via MCMC. The first two methods can be easily extended to any Λ-measure by computing the rates


$$\lambda_{b,k}=\int_{0}^{1}x^{k-2}(1-x{)}^{b-k}\Lambda (dx)$$

for each b≥k≥2. Our implementations in phylodyn through the function BNPR_Lambda() allow the user to supply any discrete or continuous probability measure Λ(dx) on [0,1]. As an example, in figure 9, we show estimation of $N_{e}(t)$ of human influenza A in New York^3^ under four different models: Kingman’s coalescent, Beta-coalescent with α=1.5, a discrete probability mass function taking values 0.2,0.7,0.9,1 with equal probability and a truncated standard normal distribution. We choose to focus on the Beta-coalescent due to its higher applicability and nice theoretical properties.

This work expands on previous phylodynamic reconstruction methods developed for binary genealogies and implemented in phylodyn [37]. We empirically demonstrate that MCMC inference performs the best on simulated data when the underlying $N_{e}(t)$ is not constant. We also show that although coalescent times and block sizes are sufficient statistics for estimating $N_{e}(t)$ and α, α can be estimated by maximum likelihood from block size information alone. Surprisingly, this method proves quite accurate. In contrast, we show that α cannot be estimated from coalescent times alone. Although testing is out of the scope of the present article, we anticipate that a likelihood ratio test for α would work well in this setting.

Finally, our methods assume that a genealogy is available without error, or rather is presented to the practitioner. We include a small simulation example in appendix A.4 comparing parameter estimates from the true simulated genealogy versus reconstructed genealogies from simulated molecular sequences. As expected, there is larger uncertainty when genealogies are estimated, in particular in the estimation of $N_{e}(t)$. A natural future direction is to incorporate our approach into a full Bayesian approach that targets the posterior distribution of $N_{e}(t)$, α and g from molecular sequences directly. We anticipate this to be a challenging problem as the state space of multifurcating trees is much larger than the space of binary trees.

## Figures and Tables

**Figure 10.**
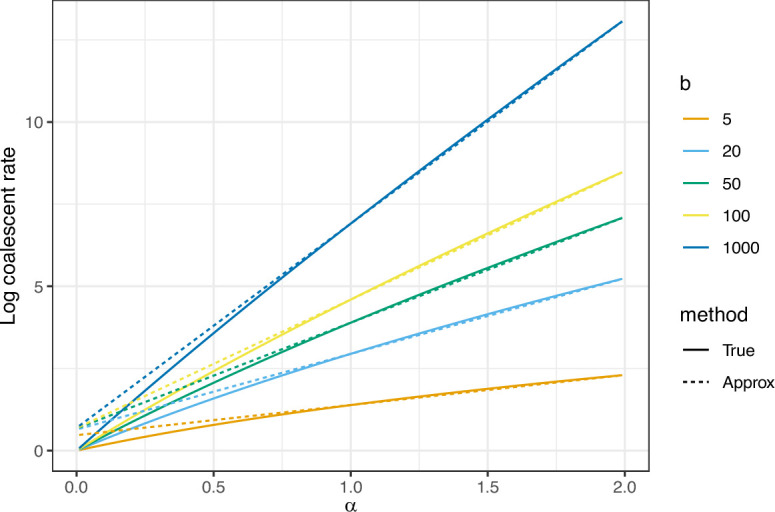
Log total coalescent rate when there are b lineages. Solid lines show the true value for different values of b and dashed lines show our approximation.

**Figure 14.**
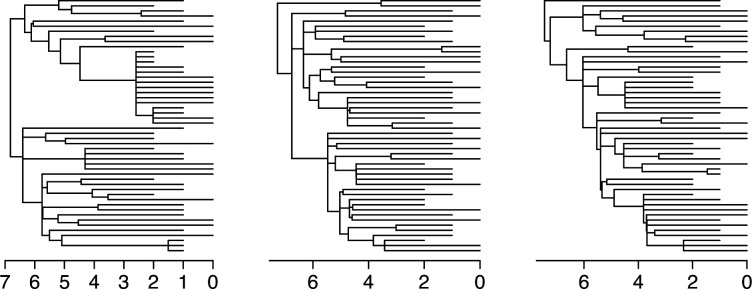
The three ’true’ genealogies used for simulation. Each was generated under a Beta-coalescent with α=1.5 and an exponential effective population size trajectory.

**Figure 15.**
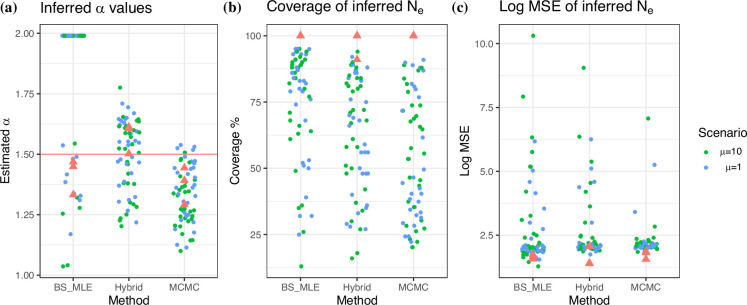
Results of inferred **α** and **$N_{e}(t)$** values under the block-size MLE, hybrid and MCMC methods based on reconstructed genealogies. (*a*) Scatterplots of inferred α values: the horizontal line is the true α=1.5. (*b*) Scatterplots of the coverage of the 95% credible intervals of the inferred $N_{e}(t)$ values. (*c*) Scatterplots of the $\log_{10}\;$ MSE of the inferred $N_{e}(t)$ values. Note the red triangles in each figure represent the value from applying our methods on the true genealogy in figure 14. The green and blue colours represent the fast mutation scenario, and the original mutation scenario, respectively.

## Data Availability

Raw data for the RSV and Enterovirus examples were downloaded from Nextstrain RSV (https://nextstrain.org/rsv/a/genome/6y) and Nextstrain Enterovirus (https://nextstrain.org/enterovirus/d68/genome) portals respectively on April 9, 2024. Raw data for the Japanese sardines were accessed from GenBank (accession nos. LC031518–LC031673), and are included here. Code can be found in the R package at [63]. The code and raw data files can also be found in [64].

## Footnotes

^1^
The data can be downloaded at https://nextstrain.org/rsv/a/G?f_country=Bangladesh,Cambodia,India,Laos,Myanmar,Nepal,Thailand,Vietnam sampled in south and southeast Asia (mainly in India and Thailand) from 1991 to 2023. Sequences were aligned with MAFFT [49] and the genealogy was reconstructed with IQ-Tree [50]. Finally, we used TreeTime [51] for temporal inference and branches of length zero were collapsed.

^2^
The data used can be downloaded at https://nextstrain.org/enterovirus/d68/genome?f_region=Europe. The analysis was performed in the same manner as the RSV example and we accessed the webpage on April 9, 2024.

^3^
Genealogy is estimated from sequences analyzed in Rambaut *et al.* [62] and available in phylodyn.

## Appendix A

### A.1. Upper bound on the total coalescent rate

***Proposition 1.*** *The total coalescent rate $\lambda_{b}$ of the Beta-coalescent is upper-bounded by*

(A 1)
λb=∑k=2b(bk)λb,k≤(b−1)(b2)α−1.

*Proof.* We will use Wendel’s inequality [65]:

(A 2)
$$x^{1-s}\leq \frac{\Gamma (x+1)}{\Gamma (x+s)}\leq (x+s{)}^{1-s},\;\;0<s<1,x>0.$$

In addition,

$$\Gamma (2-\alpha )\Gamma (\alpha )=(1-\alpha )\Gamma (1-\alpha )\Gamma (\alpha )=\frac{(1-\alpha )\pi }{\sin((1-\alpha )\pi )}$$

which implies

$$\frac{1}{\Gamma (2-\alpha )\Gamma (\alpha )}=\frac{\sin((1-\alpha )\pi )}{(1-\alpha )\pi }\in (0,1].$$

Applying the reciprocal of Wendel’s inequality twice, we have for 2≤k≤b,

(bk)λb,k(α)=b!Γ(k−α)Γ(α+b−k)Γ(2)k!(b−k)!Γ(2−α)Γ(α)Γ(b)=b(Γ(k−2+2−α)kΓ(k))(Γ(b−k+1+(α−1))(b−k+1)Γ(b−k+2))1Γ(2−α)Γ(α)(∗)≤b×(k−2)1−αk(k−1)×(b−k+1)α−1≤b×(k−1)1−αk(k−1)×(b−(k−1))α−1=b×1k(k−1)×(bk−1−1)α−1≤b×1k(k−1)×(b2)α−1

In (∗), we assumed 1<α<2. A similar proof will work for 0<α<1. Since $\sum_{k=2}^{b}\frac{1}{k(k-1)}=\frac{b-1}{b}$, we obtain the desired upper bound on the total coalescent rate when there are b lineages:

(A 3)
λb=∑k=2b(bk)λb,k≤(b−1)(b2)α−1.

Figure 10 shows that for α∈[1,2), the upper bound is a very good approximation. There may be some numerical imprecisions in the calculation of $\lambda_{b}$.∎

## A.2. Definition of performance measures

The performance measures for evaluating estimates of the effective population size are calculated as follows:

$$\begin{array}{rl} \text{Coverage} & =\frac{1}{D}\sum_{i=1}^{D}1({\hat{N}}_{e}^{H}(t_{i})>N_{e}^{T}(t_{i})>{\hat{N}}_{e}^{L}(t_{i}))\\ \text{Bias} & =\frac{1}{D}\sum_{i=1}^{D}\frac{{\hat{N}}_{e}(t_{i})-N_{e}^{T}(t_{i})}{N_{e}^{T}(t_{i})}\\ \text{Deviance} & =\frac{1}{D}\sum_{i=1}^{D}\frac{|{\hat{N}}_{e}(t_{i})-N_{e}^{T}(t_{i})|}{N_{e}^{T}(t_{i})}\\ \text{MSE} & =\frac{1}{D}\sum_{i=1}^{D}\frac{({\hat{N}}_{e}(t_{i})-N_{e}^{T}(t_{i}){)}^{2}}{N_{e}^{T}(t_{i})} \end{array}$$

where ${\hat{N}}_{e}(t)$ denotes the estimated trajectory, ${\hat{N}}_{e}^{H}(t),{\hat{N}}_{e}^{L}(t)$ the upper and lower bounds of the credible regions, respectively, and $N_{e}^{T}(t)$ the true trajectory, with grid points $\left\{t_{i}:i=1,...,D\right\}$.

## A.3. Results from simulations

Tables 2–4 and figures 11, 12 and 13 show the main results from the simulations carried out in §4.

**Table 2.** Performance measures for estimating $N_{e}(t)$ of the tree in figure 3. Values were calculated by discretizing $N_{e}(t)$ at 100 grid points.

	true α	BS MLE	hybrid	MCMC
coverage	100%	100%	95%	100%
bias	0.003	0.366	1.027	0.217
deviance	0.266	0.49	1.094	0.349
MSE	6.491	40.908	315.304	12.783

**Table 3.** Estimation of α from tree topology only. Mean ${\hat{\alpha }}^{BS}$, mean bias and MSE based on 1000 simulations for each combination of α and n.

	n	mean	bias	MSE
α=0.2	50	0.19	−0.01	0.02
	100	0.18	−0.02	0.01
	500	0.18	−0.02	0.01
α=0.5	50	0.46	−0.04	0.04
	100	0.47	−0.03	0.03
	500	0.47	−0.03	0.01
α=1	50	0.97	−0.03	0.09
	100	0.98	−0.03	0.03
	500	0.99	−0.01	0.01
α=1.5	50	1.48	−0.02	0.03
	100	1.5	−0.008	0.01
	500	1.5	−0.003	0.002
α=1.8	50	1.8	−0.005	0.01
	100	1.8	0.001	0.001
	500	1.8	−0.002	0.001

**Table 4.** Percentage of simulations where each method exhibits the lowest deviance for estimating α, where we simulated across different n and α values, sampling schedules and effective population size trajectories.

trajectory	BS MLE	hybrid	MCMC
uniform	26.6%	32.3%	41.1%
exponential	30.6%	28.5%	40.9%
boombust	32.7%	26.6%	40.7%

## A.4. Simulation study on reconstructed phylogenies

Our methods assume a multifurcating genealogy is given without error. In practice, genealogies are estimated with uncertainty from observed molecular data. To analyse the sensitivity of our estimates to genealogical estimation uncertainty, we generated three multifurcating trees with 50 tips under a Beta(2−α,α) coalescent with α=1.5 and an exponential effective population size trajectory. The tips were heterochronously sampled at t=0,1,2 with a (40%,40%,20%) split. For each of the trees shown in figure 14, we simulated 10 datasets of 50 sequences with 1000 base pairs using AliSim under the Jukes-Cantor mutation model [66]. Then, we used IQ-Tree and TreeTime to reconstruct dated, multifurcating trees via maximum likelihood [53,54] and applied our three methods to estimate α and $N_{e}(t)$ on these reconstructed genealogies. We also carried out the same analysis pipeline on datasets simulated with a 10 times faster mutation rate. Results are presented in figure 15 below. As expected, estimates of α from inferred genealogies are noisier than those obtained from the true genealogies, however inference of $N_{e}(t)$ is generally much worse when genealogies are inferred. We conclude that our methods, like any method that assumes a fixed estimated genealogy, are not robust to genealogical misestimations. A joint method for estimating the genealogy and model parameters is still an open problem.

## References

[1] Kingman JFC. 1982 The coalescent. Stoch. Process. Their Appl. **13**, 235–248. (10.1016/0304-4149(82)90011-4)

[2] Slatkin M. 2001 Simulating genealogies of selected alleles in a population of variable size. Genet. Res. **78**, 49–57. (10.1017/s0016672301005183) 11556137

[3] Wakeley J. 2009 Coalescent theory: an introduction. Greenwood Village, CO: Roberts and Co.

[4] Menardo F, Gagneux S, Freund F. 2021 Multiple merger genealogies in outbreaks of Mycobacterium tuberculosis. Mol. Biol. Evol. **38**, 290–306. (10.1093/molbev/msaa179) 32667991 PMC8480183

[5] Li LM, Grassly NC, Fraser C. 2017 Quantifying transmission heterogeneity using both pathogen phylogenies and incidence time series. Mol. Biol. Evol. **34**, 2982–2995. (10.1093/molbev/msx195) 28981709 PMC5850343

[6] Eldon B, Stephan W. 2018 Evolution of highly fecund haploid populations. Theor. Popul. Biol. **119**, 48–56. (10.1016/j.tpb.2017.10.002) 29111301

[7] Eldon B. 2020 Evolutionary genomics of high fecundity. Annu. Rev. Genet. **54**, 213–236. (10.1146/annurev-genet-021920-095932) 32870729

[8] Niwa HS, Nashida K, Yanagimoto T. 2016 Reproductive skew in Japanese sardine inferred from DNA sequences. ICES J. Mar. Sci. **73**, 2181–2189. (10.1093/icesjms/fsw070)

[9] Sargsyan O, Wakeley J. 2008 A coalescent process with simultaneous multiple mergers for approximating the gene genealogies of many marine organisms. Theor. Popul. Biol. **74**, 104–114. (10.1016/j.tpb.2008.04.009) 18554676

[10] Byeon SY, Oh HJ, Kim S, Yun SH, Kang JH, Park SR, Lee HJ. 2019 The origin and population genetic structure of the ‘golden tide’ seaweeds, Sargassum horneri, in Korean waters. Sci. Rep. **9**, 7757. (10.1038/s41598-019-44170-x) 31123297 PMC6533256

[11] Der R, Epstein C, Plotkin JB. 2012 Dynamics of neutral and selected alleles when the offspring distribution is skewed. Genetics **191**, 1331–1344. (10.1534/genetics.112.140038) 22661323 PMC3416011

[12] Eldon B, Stephan W. 2024 Sweepstakes reproduction facilitates rapid adaptation in highly fecund populations. Mol. Ecol. **33**, e16903. (10.1111/mec.16903) 36896794

[13] Árnason E, Koskela J, Halldórsdóttir K, Eldon B. 2023 Sweepstakes reproductive success via pervasive and recurrent selective sweeps. eLife **12**, e80781. (10.7554/elife.80781) 36806325 PMC9940914

[14] Wakeley J. 2003 Gene genealogies when the sample size exceeds the effective size of the population. Mol. Biol. Evol. **20**, 208–213. (10.1093/molbev/msg024) 12598687

[15] Möhle M, Sagitov S. 2003 Coalescent patterns in diploid exchangeable population models. J. Math. Biol. **47**, 337–352. (10.1007/s00285-003-0218-6) 14523577

[16] Pitman J. 1999 Coalescents with multiple collisions. Ann. Probab. **27**, 1870–1902. (10.1214/aop/1022874819)

[17] Sagitov S. 1999 The general coalescent with asynchronous mergers of ancestral lines. J. Appl. Probab. **36**, 1116–1125. (10.1017/s0021900200017903)

[18] Berestycki J, Berestycki N, Schweinsberg J. 2007 Beta-coalescents and continuous stable random trees. Ann. Probab. 1835–87 **35**. (10.1214/009117906000001114)

[19] Eldon B, Wakeley J. 2006 Coalescent processes when the distribution of offspring number among individuals is highly skewed. Genetics **172**, 2621–2633. (10.1534/genetics.105.052175) 16452141 PMC1456405

[20] Schweinsberg J. 2003 Coalescent processes obtained from supercritical Galton–Watson processes. Stoch. Process. Their Appl. **106**, 107–139. (10.1016/s0304-4149(03)00028-0)

[21] Berestycki N. 2009 Recent progress in coalescent theory. arXiv. (10.48550/arXiv.0909.3985)

[22] Hoscheit P, Pybus OG. 2019 The multifurcating skyline plot. Virus Evol. **5**, vez031. (10.1093/ve/vez031) 31528357 PMC6736156

[23] Korfmann K, Sellinger TPP, Freund F, Fumagalli M, Tellier A. 2024 Simultaneous inference of past demography and selection from the ancestral recombination graph under the Beta coalescent. Peer Community J **4**, 397. (10.24072/pcjournal.397)

[24] Eldon B, Birkner M, Blath J, Freund F. 2015 Can the site-frequency spectrum distinguish exponential population growth from multiple-merger coalescents? Genetics **199**, 841–856. (10.1534/genetics.114.173807) 25575536 PMC4349076

[25] Koskela J. 2018 Multi-locus data distinguishes between population growth and multiple merger coalescents. Stat. Appl. Genet. Mol. Biol. **17**. (10.1515/sagmb-2017-0011) 29897888

[26] Matuszewski S, Hildebrandt ME, Achaz G, Jensen JD. 2018 Coalescent processes with skewed offspring distributions and nonequilibrium demography. Genetics **208**, 323–338. (10.1534/genetics.117.300499) 29127263 PMC5753866

[27] Koskela J, Wilke Berenguer M. 2019 Robust model selection between population growth and multiple merger coalescents. Math. Biosci. **311**, 1–12. (10.1016/j.mbs.2019.03.004) 30851276

[28] Freund F, Siri-Jégousse A. 2021 The impact of genetic diversity statistics on model selection between coalescents. Comput. Stat. Data Anal. **156**, 107055. (10.1016/j.csda.2020.107055)

[29] Miró Pina V, Joly É, Siri-Jégousse A. 2023 Estimating the Lambda measure in multiple-merger coalescents. Theor. Popul. Biol. **154**, 94–101. (10.1016/j.tpb.2023.09.002) 37742787

[30] Ho SYW, Shapiro B. 2011 Skyline‐plot methods for estimating demographic history from nucleotide sequences. Mol. Ecol. Resour. **11**, 423–434. (10.1111/j.1755-0998.2011.02988.x) 21481200

[31] Palacios JA, Minin VN. 2012 Integrated nested Laplace approximation for Bayesian nonparametric phylodynamics. In Proc. 28th Conf. on Uncertainty in Artificial Intelligence, pp. 726–735. Arlington, VA: AUAI Press.

[32] Lan S, Palacios JA, Karcher M, Minin VN, Shahbaba B. 2015 An efficient Bayesian inference framework for coalescent-based nonparametric phylodynamics. Bioinformatics **31**, 3282–3289. (10.1093/bioinformatics/btv378) 26093147 PMC4795633

[33] Felsenstein J, Rodrigo AG. 1999 Coalescent approaches to HIV population genetics. In The evolution of HIV (ed. KA Crandall), pp. 233–272. Baltimore, MD: Johns Hopkins University Press.

[34] Wakeley J, Sargsyan O. 2009 Extensions of the coalescent effective population size. Genetics **181**, 81. (10.1534/genetics.108.092460) 19001293 PMC2621185

[35] Freund F. 2020 Cannings models, population size changes and multiple-merger coalescents. J. Math. Biol. **80**, 1497–1521. (10.1007/s00285-020-01470-5) 32008102 PMC7052052

[36] Rue H, Held L. 2005 Gaussian Markov random fields: theory and applications. New York, NY: Chapman Hall/CRC.

[37] Karcher MD, Palacios JA, Lan S, Minin VN. 2017 phylodyn: an R package for phylodynamic simulation and inference. Mol. Ecol. Resour. **17**, 96–100. (10.1111/1755-0998.12630) 27801980 PMC5466693

[38] Rue H, Martino S, Chopin N. 2009 Approximate Bayesian inference for latent Gaussian models by using integrated nested Laplace approximations. J. R. Stat. Soc. Ser. B **71**, 319–392. (10.1111/j.1467-9868.2008.00700.x)

[39] Hadfield J, Megill C, Bell SM, Huddleston J, Potter B, Callender C, Sagulenko P, Bedford T, Neher RA. 2018 Nextstrain: real-time tracking of pathogen evolution. Bioinformatics **34**, 4121–4123. (10.1093/bioinformatics/bty407) 29790939 PMC6247931

[40] Benson DA, Cavanaugh M, Clark K, Karsch-Mizrachi I, Lipman DJ, Ostell J, Sayers EW. 2012 GenBank. Nucleic Acids Res. **45**, D36. (10.1093/nar/gkw1070) PMC3531190 23193287

[41] Li Y *et al*. 2022 Global, regional, and national disease burden estimates of acute lower respiratory infections due to respiratory syncytial virus in children younger than 5 years in 2019: a systematic analysis. Lancet **399**, 2047–2064. (10.1016/s0140-6736(22)00478-0) 35598608 PMC7613574

[42] Divarathne MVM, Ahamed R, Noordeen F. 2019 The impact of RSV-associated respiratory disease on children in Asia. J. Pediatr. Infect. Dis. **14**, 79–88. (10.1055/s-0038-1637752) 32300274 PMC7117084

[43] Pebody R *et al*. 2020 Approaches to use the WHO respiratory syncytial virus surveillance platform to estimate disease burden. Influenza Other Respir. Viruses **14**, 615–621. (10.1111/irv.12667) 31595655 PMC7578280

[44] Weber MW, Weber MW, Mulholland EK, Mulholland EK, Greenwood BM. 1998 Respiratory syncytial virus infection in tropical and developing countries. Trop. Med. Int. Health **3**, 268–280. (10.1046/j.1365-3156.1998.00213.x) 9623927

[45] Thongpan I, Vongpunsawad S, Poovorawan Y. 2020 Respiratory syncytial virus infection trend is associated with meteorological factors. Sci. Rep. **10**, 10931. (10.1038/s41598-020-67969-5) 32616819 PMC7331681

[46] Griffiths C, Drews SJ, Marchant DJ. 2017 Respiratory syncytial virus: infection, detection, and new options for prevention and treatment. Clin. Microbiol. Rev. **30**, 277–319. (10.1128/cmr.00010-16) 27903593 PMC5217795

[47] Falsey AR, Walsh EE. 2000 Respiratory syncytial virus infection in adults. Clin. Microbiol. Rev. **13**, 371–384. (10.1128/cmr.13.3.371-384.2000) 10885982 PMC88938

[48] Jha A, Jarvis H, Fraser C, J.M. Openshaw P. 2016 Respiratory syncytial virus. In SARS, MERS and other viral lung infections (eds DS Hui, GA Rossi, SL Johnston), pp. 84–109. Sheffield, UK: European Respiratory Society. (10.1183/2312508x.10010315) 28742304

[49] Katoh K, Misawa K, Kuma K, Miyata T. 2002 MAFFT: a novel method for rapid multiple sequence alignment based on fast Fourier transform. Nucleic Acids Res. **30**, 3059–3066. (10.1093/nar/gkf436) 12136088 PMC135756

[50] Nguyen LT, Schmidt HA, von Haeseler A, Minh BQ. 2015 IQ-TREE: a fast and effective stochastic algorithm for estimating maximum-likelihood phylogenies. Mol. Biol. Evol. **32**, 268–274. (10.1093/molbev/msu300) 25371430 PMC4271533

[51] Sagulenko P, Puller V, Neher RA. 2018 TreeTime: maximum-likelihood phylodynamic analysis. Virus Evol. **4**, vex042. (10.1093/ve/vex042) 29340210 PMC5758920

[52] Dyda A, Stelzer-Braid S, Adam D, Chughtai AA, MacIntyre CR. 2018 The association between acute flaccid myelitis (AFM) and Enterovirus D68 (EV-D68)—what is the evidence for causation? Eurosurveillance **23**. (10.2807/1560-7917.es.2018.23.3.17-00310) PMC5792700 29386095

[53] Moline H, Kalaskar A, Pomputius WF III, Lopez A, Routh J, Kenyon C, Griffith J. 2019 Six cases of acute flaccid myelitis in children—Minnesota, 2018. MMWR Morb. Mortal. Wkly Rep. **68**, 356–358. (10.15585/mmwr.mm6815a4) 30998669 PMC6476059

[54] Holm-Hansen CC, Midgley SE, Fischer TK. 2016 Global emergence of enterovirus D68: a systematic review. Lancet Infect. Dis. **16**, e64–e75. (10.1016/s1473-3099(14)70968-5) 26929196

[55] Centers for Disease Control and Prevention. 2011 Clusters of acute respiratory illness associated with human enterovirus 68—Asia, Europe, and United States, 2008–2010. MMWR Morb. Mortal. Wkly Rep. **60**, 1301–1304. (10.15585/mmwr.mm6812a1) 21956405

[56] McKay SL *et al*. 2018 Increase in acute flaccid myelitis—United States, 2018. MMWR Morb. Mortal. Wkly Rep. **67**, 1273–1275. (10.15585/mmwr.mm6745e1) 30439867 PMC6290805

[57] Benschop KS *et al*. 2021 Re-emergence of enterovirus D68 in Europe after easing the COVID-19 lockdown, September 2021. Eurosurveillance **26**, 2100998. (10.2807/1560-7917.es.2021.26.45.2100998) 34763750 PMC8646978

[58] Wang G, Zhuge J, Huang W, Nolan SM, Gilrane VL, Yin C, Dimitrova N, Fallon JT. 2017 Enterovirus D68 subclade B3 strain circulating and causing an outbreak in the United States in 2016. Sci. Rep. **7**, 1242. (10.1038/s41598-017-01349-4) 28455514 PMC5430842

[59] Birkner M, Blath J, Steinrücken M. 2011 Importance sampling for Lambda-coalescents in the infinitely many sites model. Theor. Popul. Biol. **79**, 155–173. (10.1016/j.tpb.2011.01.005) 21296095 PMC3085644

[60] Sukumaran S, Sebastian W, Gopalakrishnan A. 2016 Population genetic structure of Indian oil sardine, Sardinella longiceps along Indian coast. Gene **576**, 372–378. (10.1016/j.gene.2015.10.043) 26494160

[61] Drummond A, Rodrigo AG. 2000 Reconstructing genealogies of serial samples under the assumption of a molecular clock using serial-sample UPGMA. Mol. Biol. Evol. **17**, 1807–1815. (10.1093/oxfordjournals.molbev.a026281) 11110896

[62] Rambaut A, Pybus OG, Nelson MI, Viboud C, Taubenberger JK, Holmes EC. 2008 The genomic and epidemiological dynamics of human influenza A virus. Nature **453**, 615–619. (10.1038/nature06945) 18418375 PMC2441973

[63] Palacios J. 2024 phylodyn. GitHub. https://github.com/JuliaPalacios/phylodyn

[64] Zhang J, Palacios J. 2024 Data from: Multiple merger coalescent inference of effective population size. Zenodo. https://zenodo.org/records/1399928810.1098/rstb.2023.030639976412

[65] Qi F, Luo QM. 2013 Bounds for the ratio of two gamma functions: from Wendel’s asymptotic relation to Elezović-Giordano-Pečarić’s theorem. J. Inequalities Appl. **2013**, 20. (10.1186/1029-242x-2013-542)

[66] Ly-Trong N, Barca GMJ, Minh BQ. 2023 AliSim-HPC: parallel sequence simulator for phylogenetics. Bioinformatics **39**, btad540. (10.1093/bioinformatics/btad540) 37656933 PMC10534053

